# Chorioamnionitis secondary to *Ureaplasma parvum* infection: a case report

**DOI:** 10.1515/almed-2023-0004

**Published:** 2023-02-15

**Authors:** Antonio Moreno-Flores, María Domínguez-Landesa, María Guadalupe Vázquez-López, Laura Sante-Fernández

**Affiliations:** Hospital Universitario Lucus Augusti, Lugo, Spain

**Keywords:** chorioamnionitis, preterm labour, *Ureaplasma*

## Abstract

**Objectives:**

*Ureaplasma* species are the most frequently isolated microorganisms in cases of spontaneous preterm labor, premature rupture of the membranes, or chorioamnionitis.

**Case presentation:**

A woman at 28^+6^ weeks of gestation with no apparent history of interest presented at the hospital with contractions. Upon suspicion of chorioamnionitis, the patient was admitted for a low segment transverse cesarean section, which was completed without any complications. The patient was discharged at 7 days. The newborn remained stable and showed no clinical signs of infection. However, on suspicion of chorioamnionitis, empirical treatment with intravenous ampicillin (2 g every 6 h) and gentamicin (5 mg/kg once daily) was initiated. Samples of pharyngeal/tonsillar, ear, and anal/rectal exudates were collected. At 24 h, all samples were positive for *Ureaplasma parvum*. Empirical treatment was suspended, and treatment with intravenous azithromycin was initiated (12 mg once daily). Endocervical and placental exudates were also positive for *U. parvum*. Fifty-two days after birth, the newborn was discharged.

**Conclusions:**

The relationship between *Ureaplasma* spp. colonization and perinatal disease seem to be clear. However, the high frequency of vaginal *Ureaplasma* spp*.* colonization and high rates of term labor among pregnant women with this colonization make further studies necessary.

## Introduction

*Ureaplasma urealyticum* and *Ureaplasma parvum* are often found in the human genital tract and are the most frequently isolated microorganisms in the amniotic fluid and placenta of women after pre-term delivery [1], [2], [3], [4], [5]. It is striking that, although there is a strong association between colonization and perinatal infections, a significant proportion of pregnant women who are colonized by these species do not develop any clinical manifestation of infection [1], [2], [3], [4].

## Case presentation

We report the case of a woman at 28^+6^ weeks of gestation without an apparent history of interest, with controlled pregnancy, who presented at the hospital with contractions. Blood analysis revealed leukocytosis (21,030/mm^3^) with 91.1% of neutrophils. On clinical suspicion of chorioamnionitis and, in view of the breech presentation of the fetus, the patient was admitted for a low-segment transverse cesarean section. Prior to surgery, empirical prophylactic treatment was administered with intravenous piperacillin/tazobactam (4 g/0.5 g/6 h) and clarithromycin (500 mg/12 h) and a single dose of cefazolin (1 g). Prior to initiation of antibiotic therapy, a sample of vaginal exudate was sent for culture. Common saprophytic flora was isolated. Surgery was completed without any complications and the patient evolved favorably. Antibiotic therapy was withdrawn after the patient had remained afebrile for 48 h. The patient was discharged at 7 days. The neonate remained stable in respiratory and hemodynamic terms and was afebrile, without any clinical evidence of infection. However, on suspicion of chorioamnionitis, empirical treatment with intravenous ampicillin (2 g every 6 h) and gentamicin (5 mg/kg once daily) was initiated. Samples of pharyngeal/tonsillar, ear, and anal/rectal exudates were collected for molecular detection of sexually-transmitted microorganisms (Allplex™ STI Essential Assay, Seegene Inc). At 24 h, all samples were positive for *U. parvum*. Empirical treatment was withdrawn, and treatment with intravenous azithromycin was initiated (12 mg once daily). Endocervical and placental exudates were also positive for *U. parvum*. Fifty-two days after birth, the newborn was discharged.

## Discussion

Chorioamnionitis is the inflammation of fetal membranes, possibly due to intra-amniotic microorganism infection [4, 6]. Although *Ureaplasma* spp. are often found in the human genital tract, they are the microorganisms most frequently linked to spontaneous preterm birth, premature rupture of the membranes or chorioamnionitis [1, 2, 5, 7]. In these cases, *U. parvum* is the most common bacterium found in the amniotic cavity [1, 3]. In the neonate, these microorganisms may cause bronchopulmonary dysplasia, necrotizing enterocolitis, premature retinopathy or brain lesions, among other clinical manifestations [1, 2, 8, 9]. Although *Ureaplasma* spp. has some virulence factors, none has been directly identified as the causative agent of preterm birth [3]. Several studies point to multiple banded antigen as a potential inducer of host immune response. This antigen induces the production of cytokines, which could favor the onset of contractions and, in case of postnatal infection, the development of bronchopulmonary dysplasia [3], [4], [5], [6, 8].

Owing to the lack of a cell wall, *Ureaplasma* spp. are highly sensitive to dehydratation and changes in temperature. They are fastidious microorganisms that require special culture methods because of their growth requirements [2]. Molecular techniques offer advantages over other methods, such a higher diagnostic sensitivity, the capacity to distinguish between species and quantify results [9]. Beta-lactam antibiotics are ineffective due to the lack of a cell wall [2, 3, 9]. There are limited, effective, non-teratogenic therapies available; being macrolides the most widely used family of antibiotics, especially azithromycin and clarithromycin [2, 3]. However, the recommended empirical therapy for chorioamnionitis is gentamicin in combination with ampicillin [2, 5], a poorly effective treatment in these cases [2, 5]. There is no clear scientific evidence either of the cost-effectiveness of active screening and eradication strategies. In addition, colonization does not necessarily induce the development of infection, and eradication treatments may elicit adverse events, increase resistance to antibiotics, and cause changes in the microbioma [1, 2].

In conclusion, the relationship between colonization by these microorganisms and perinatal disease seems to be clear. However, the high frequency of vaginal *Ureaplasma* spp. colonization and high rates of uncomplicated term birth among pregnant women with *Ureaplasma* spp*.* colonization make further studies necessary.

## Lessons learned

– Invasion of the amniotic cavity by microorganisms may have severe clinical effects on the neonate.
– Molecular techniques improve microbiological diagnosis of microorganisms that require special culture methods.
– Further studies are needed for a better understanding of this type of infections.
